# Feeding Behavior and Virus-transmission Ability of Insect Vectors Exposed to Systemic Insecticides

**DOI:** 10.3390/plants9070895

**Published:** 2020-07-15

**Authors:** Elisa Garzo, Aránzazu Moreno, María Plaza, Alberto Fereres

**Affiliations:** Departamento de Protección Vegetal, Instituto de Ciencias Agrarias (ICA), Consejo Superior de Investigaciones Científicas (CSIC), 28006 Madrid, Spain; amoreno@ica.csic.es (A.M.); mariap@ica.csic.es (M.P.); a.fereres@csic.es (A.F.)

**Keywords:** feeding behavior, aphids, whitefly, systemic insecticides, plant virus transmission, electrical-penetration-graph (EPG) technique

## Abstract

The majority of plant viruses depend on Hemipteran vectors for their survival and spread. Effective management of these insect vectors is crucial to minimize the spread of vector-borne diseases, and to reduce crop damage. The aim of the present study was to evaluate the effect of various systemic insecticides on the feeding behavior of *Bemisia tabaci* and *Myzus persicae*, as well as their ability to interfere with the transmission of circulative viruses. The obtained results indicated that some systemic insecticides have antifeeding properties that disrupt virus transmission by their insect vectors. We found that some of the tested insecticides significantly reduced phloem contact and sap ingestion by aphids and whiteflies, activities that are closely linked to the transmission of phloem-limited viruses. These systemic insecticides may play an important role in reducing the primary and secondary spread of tomato yellow leaf curl virus (TYLCV) and turnip yellows virus (TuYV), transmitted by *B. tabaci* and *M. persicae*, respectively.

## 1. Introduction

Viruses are obligate parasites that use host-cell machinery to produce their progeny [1]. Several taxonomic groups of phytophagous insects may act as vectors of plant viruses [2]. Hemipteran insects are the most important and numerous vectors of plant viruses, being able to transmit more than 70% of all known insect-borne viruses. Among these, aphids (Hemiptera: Aphididae) and whiteflies (Hemiptera: Aleyrodidae) are major vectors of plant viruses, transmitting more than 500 virus species [3]. Plant viruses were classified in two categories by Kennedy et al. [4], and Harris [5] depending on their ability to circulate through the body of their insect vectors, noncirculative (NC) and circulative viruses (CVs). NC virus particles attach to the cuticle of their vectors for a short period of time without any circulation within the vector’s body [6]. This category is divided into two subcategories depending on the duration of virus retention in the vector, nonpersistent (NP) and semipersistent (SP) [7]. Insect vectors quickly transmit NP viruses after fast exploratory superficial probes. These NP viruses persist for a few hours in the vector, are acquired and inoculated during brief (seconds) intracellular stylet punctures in the epidermis and mesophyll cells, visualized as standard potential drops (pd) by the electrical-penetration-graph (EPG) technique [8,9,10]. Furthermore, vectors can transmit NP viruses immediately after acquiring the virus without any latent period. However, SP viruses can persist many hours or days in their vectors, and need several hours for acquisition and inoculation, with no latent period [11]. When the insect vector moults, the transmissibility of both NP and SP viruses is lost. The second category corresponds to circulative viruses (CVs), also frequently referred to as persistent (P) viruses. Some CVs propagate in their insect vector and are therefore termed CV-propagative. However, some circulative viruses, such as pea enation mosaic virus (PEMV), can be inoculated in epidermal cells during brief intracellular stylet punctures by aphids, similar to nonpersistent viruses [10]. At the same time, the virus needs to circulate through the insect’s body, crossing the gut and salivary-gland membranes [12]. These viruses need a latent period of several hours or days to circulate inside the insect’s body before they can be transmitted. CV particles are ingested generally from phloem sieve elements, internalized by the insect vector, transported across gut cellular membranes, carried by the hemolymph, and enter the salivary glands. Lastly, saliva transports virus particles through the salivary duct to reach the phloem cells of an uninfected host plant [3,13]. 

Insects of the Hemiptera order transmit most plant viruses and many other plant pathogens. Most virus vectors include aphids, whiteflies, mealybugs, and leafhoppers that transmit viruses such as the citrus tristeza virus (CTV) or tomato yellow leaf curl virus (TYLCV), both causing economically-important emerging diseases. Psyllids, sharpshooters, and spittlebugs are vectors of bacteria that cause devastating diseases such as huanglongbing (HLB), Pierce’s disease (PD) or olive quick decline syndrome (OQDS). Vector-borne plant pathogens are transmitted from plant to plant after specific behavioral events in which insect vectors find, land on, probe, and feed on an infected plant. Then, infective insects need to search, find, and feed on a noninfected plant to complete the transmission process [14]. The probing and feeding behavior of piercing and sucking insects can be monitored using the EPG technique [15], which is a useful tool to investigate insect-plant interactions, including the localization and characterization of host-plant resistance to aphids [16,17,18,19]. Furthermore, EPG allows to understand the transmission mechanisms of plant pathogens by their insect vectors [11,20,21,22,23] and to study the mode of action of insecticides [24,25,26,27,28,29,30,31,32]. 

**Figure 1:**
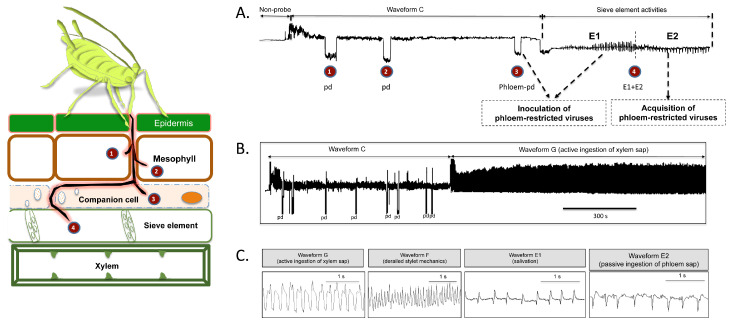
Electrical-penetration-graph (EPG) recording of green peach (*Myzus persicae*) on *Physalis floridana* Rydb. (**A**) Overview of electrical-penetration graphs in which different numbers indicate specific EPG patterns where the aphid inserts stylets into a specific cell. (**1**,**2**) Potential drops (pd) where the aphids insert their stylets in mesophyll cells. (**3**) Phloem-pd; aphid inserts stylets in companion or sieve element cells. (**4**) Stylets penetrate a sieve element. Patterns (**3**) and (**4**) are involved in the transmission of phloem-restricted viruses. Inoculation of phloem-limited viruses occurs during phloem-pd and E1 waveforms while the acquisition occurs during Waveform E2. np, nonprobing behavior; C, intercellular apoplastic stylet pathway; pd, intracellular punctures; phloem-pd, brief phloem punctures on companion or sieve elements cells. (**B**) EPG recording showing intercellular apoplastic stylet pathway (Waveform C), including some pds, and ending on active intake of xylem sap (Waveform G). (**C**) Detail of specific waveforms. G, active intake of xylem sap; F, derailed stylet mechanics; E1, salivation into phloem sieve elements; E2, passive phloem sap uptake from sieve elements.

When insects such as aphids or whiteflies insert their stylets into the plants, the circuit is closed, and EPG signals are recorded on a computer. A series of characteristic EPG waveforms are associated with stylet tip positions inside the plant tissue, and with specific probing and feeding activities, as illustrated in Figure 1 [11,22,33,34,35,36,37,38]. Waveform NP is a flat line associated to nonprobing behavior (no stylet contacts with leaf tissue), and Waveform C represents the intercellular apoplastic stylet pathway where insects show cyclic activity of mechanical stylet penetration and saliva secretion. Waveform potential drop (pd) represents an intracellular stylet puncture. Two different pds were described, the standard pd and the phloem-pd, the latter of which is associated with stylet punctures in sieve elements and companion cells. Two waveforms related to long-term phloem-phase activities were described: Waveform E1, which represents salivation into phloem sieve elements at the beginning of the phloem phase, and Waveform E2, which is correlated with passive phloem sap uptake from the sieve elements. Furthermore, Waveform G represents the active intake of xylem sap, and Waveform F represents derailed stylet mechanics. “Probe” refers to any type of event during the period in which the stylets of an individual insect are located in the plant tissue, and “no-probe” refers to the event with no waveform (indicating that the insect has its stylets outside of the plant tissue).

The feeding behavior of whiteflies and aphids has many similarities. Both are obligate phloem feeders that occasionally ingest xylem sap. In both insect families, the phloem phase begins with the penetration of a sieve element by maxillary stylet tips, followed by the secretion of watery saliva into the sieve element (Waveform E1), and then passively ingesting phloem sap from the sieve element (Waveform E2), that frequently occurs for hours [39]. There are also some important differences in the feeding behavior of both groups, principally regarding the frequency of brief intracellular stylet punctures (standard pds). Whiteflies produce far fewer pds [38,40], while aphids typically make many more pds during the stylet pathway phase [34,41]. Recently, Jimenez et al. [11] described a new type of pd named phloem-pd that represents key stylet activity in plant cells associated with the transmission of phloem-limited virus by aphids. A single brief phloem-pd (3–5 s) was the behavioral event leading to the transmission of both semipersistent (SP) (11,22) and persistent (P) (23) phloem-limited viruses by aphids. 

Sweet potato whitefly *Bemisia tabaci* (Gennadius) and green peach aphid *Myzus persicae* (Sulzer) are serious global agricultural pests, causing severe damage by direct feeding and, more importantly, by their ability to transmit a large number of plant viruses [42,43,44]. *Bemisia tabaci* transmits a large number of CVs within the Geminiviridae family (e.g., TYLCV). Furthermore, green peach aphid *Myzus persicae* is responsible for the transmission of over 100 viruses [45]. Aphid stylet activities in the phloem are involved in the transmission of phloem-restricted luteoviruses (e.g., TuYV). [35]. 

The effective management of the insect vectors of plant viruses is crucial to minimizing the spread of plant viruses, thus reducing crop damage. Control measures against vectors and vector activities can be grouped into four classes: (1) reducing vector populations, (2) reducing virus sources, (3) interference with vector landing, and (4) interference with the transmission process [3]. New-generation systemic insecticides may be used as part of an integrated pest-management (IPM) program, which can include biological, cultural, and genetic practices. Systemic insecticides play an important role in managing vector populations by reducing the number of insects that are able to acquire and transmit the virus, but also by altering the feeding behavior linked with the transmission of plant pathogens [32,46,47,48]. Appropriate insecticides may provide an effective way to control viruses transmitted both in a semipersistent and persistent mode [47,49]. However, they are usually ineffective in preventing the spread of NP viruses because an aphid vector needs extremely short feeding time (seconds) to transmit NP viruses from plant to plant. In some cases, some insecticides can also contribute to the spread of NP virus diseases by inducing greater vector activity and mobility, which results in more inoculation attempts than when insects are in a calmer state [46,50,51,52]. Moreover, one of the main current problems is the development of resistances to most insecticide groups by insect vectors, which make their control even more difficult [31,53,54,55]. 

Thus, the aim of the present study was to evaluate the effect of various systemic insecticides on the feeding behavior of *B. tabaci* and *M. persicae,* and on their ability to interfere with the transmission of TYLCV and TuYV, respectively. Both viruses are transmitted in a circulative persistent mode and cause serious global losses to horticultural crops. Cyantraniliprole, flupyradifurone, and pymetrozine are systemic insecticides that showed to be effective in controlling different stages of whitefly *B. tabaci,* and are able to reduce the ability to transmit plant viruses because they induce rapid feeding cessation [30,49,56,57,58]. In the same way, sulfoxaflor is highly effective against a wide range of sap-feeding insects, especially against aphids. This insecticide acts as a nicotinic acetylcholine receptor (nAChR) competitive modulator, and it provokes feeding cessation of sap-sucking insects [31]. Flonicamid is very effective against aphids and disrupts the feeding behavior of hemipteran insects very quickly, regardless of differences in species, stages, and morphs [59]. Spirotetramat is a systemic insecticide with phloem and xylem mobility that acts as a lipid-biosynthesis inhibitor, reducing the fecundity and fertility of sap-sucking insects such as aphids, psyllids, scales, leafminers, thrips, mealybugs, and whiteflies [60].

The nontarget effects of insecticides in natural enemies and pollinators are actually regulated by a detailed complex of actions to prevent undesirable environmental risks. In IPM programs, chemical control should be selective towards the target pest species and ideally harmless towards nontarget beneficial organisms [61,62]. Several studies evaluated the toxicity of the insecticides used in the present work on nontarget organisms [63,64,65]. Some of these chemical compounds may have undesirable effects on specific biocontrol agents; therefore, their inclusion in sustainable IPM programs should be adopted with caution. 

## 2. Results

### 2.1. Effect of Different Systemic Insecticides on Probing and Feeding Behavior of Bemisia tabaci 

**Figure 2:**
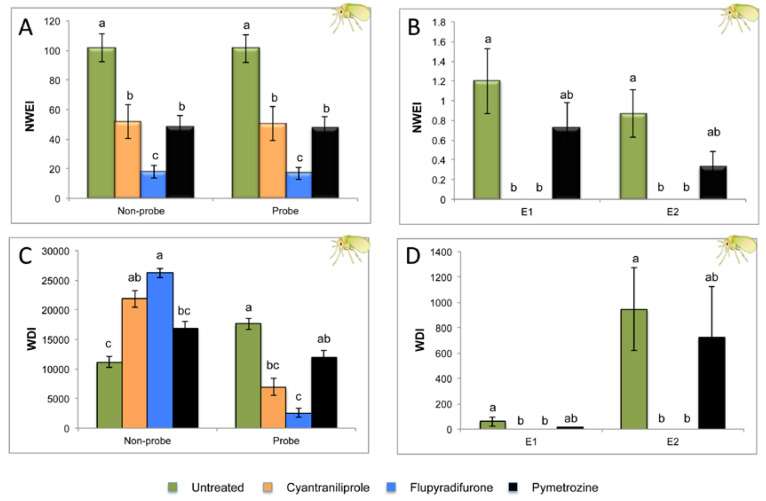
Nonsequential EPG variables (mean ± SE) for whiteflies exposed to tomato plants treated with systemic insecticides (cyantraniliprole, flupyradifurone, and pymetrozine) and untreated plants. (**A**,**B**) Number of waveform events per insect (NWEI); (**C**,**D**) Waveform duration per insect (WDI; time expressed in seconds). Waveform nonprobe, no stylet contacts with leaf tissue; Probe, all activities into plant tissue in which stylets were involved (Waveforms C, pds, F, G, E1, and E2); Waveform E1, salivation in sieve element; Waveform E2, passive phloem sap uptake from sieve elements. Mean values followed by different letters were significantly different (*P* ≤ 0.05) according to Dunn’s test with Bonferroni correction.

The effect of cyantraniliprole, flupyradifurone, and pymetrozine on the probing and feeding behavior of *B. tabaci* is shown in Figure 2 and Table 1. The number (NWEI) and total duration (WDI) of probing and phloem activities (salivation and passive phloem sap ingestion into sieve elements) were significantly reduced or suppressed on plants treated with cyantraniliprole and flupyradifurone compared to the untreated control (Figure 2). This behavior was even more remarkable in the case of flupyradifurone. However, the total duration of the probe and the phloem activities of whiteflies exposed to pymetrozine-treated plants were not significantly reduced when compared with the untreated control (Figure 2C,D). Total probing duration was much shorter on whiteflies exposed to flupyradifurone than to pymetrozine-treated plants (Figure 2C). Whiteflies started probing with no significant delay (H = 1.146; df = 3; *P* = 0.766) when they were exposed to insecticide-treated plants and to untreated control plants (Table 1; time to first probe from start of EPG). Furthermore, whiteflies were unable to show any phloem activity (Waveform E) during the eight hours of recording when plants were treated with cyantraniliprole and flupyradifurone (proportion of individuals that produced the waveform type (PPW) Waveform E: 0 out of 15 for both insecticides) as opposed to whiteflies exposed to untreated control plants (PPW Waveform E: 9 out of 15) and pymetrozine (PPW Waveform E: 7 out of 15). In consequence, the time from first probe to first phloem activity (E) was significantly longer (H = 13.911; df = 3; *P* = 0.003) for whiteflies feeding on cyantraniliprole and flupyradifurone-treated plants than those exposed to untreated control plants (Table 1). The number of whiteflies that showed a passive phloem ingestion phase (E2) was lower on pymetrozine-treated (PPW waveform E2: 3 out of 15) than on untreated control plants (PPW Waveform E2: 6 out of 15), but no significant differences were observed (χ^2^ = 1.429; *P* = 0.427). No significant differences were observed on the percentage of time spent in probing nor in phloem activities between whiteflies feeding on pymetrozine-treated and on untreated plants.

### 2.2. Effect of Different Systemic Insecticides on Probing and Feeding Behavior of Myzus persicae 

**Figure 3:**
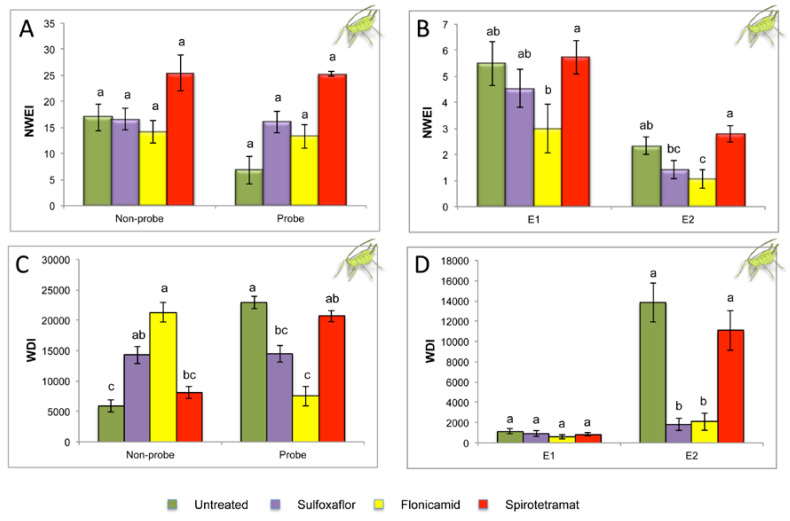
Nonsequential EPG variables (mean ± SE) for aphids exposed to *Physallis floridana* treated with systemic insecticides (sulfoxaflor, flonicamid, and spirotetramat) and to untreated plants. (**A**,**B**) Number of waveform events per insect (NWEI); (**C**,**D**) Waveform duration per insect (WDI; time expressed in seconds). Waveform nonprobe, no stylet contacts with leaf tissue; Probe, all activities into plant tissue in which stylets were involved (Waveforms C, pds, F, G, E1, and E2); Waveform E1, salivation in sieve element; Waveform E2, passive phloem sap uptake from sieve elements. Mean values followed by different letters were significantly different (*P* ≤ 0.05) according to Dunn’s test with Bonferroni correction.

The effect of sulfoxaflor, flonicamid, and spirotetramat on the probing and feeding behavior of *M. persicae* is shown in Figure 3 and Table 2. No significant differences were observed in the number of waveform events per insect (NWEI) of the nonprobe (H = 6.705, df = 3, *P* = 0.082) and probe (H = 7.673, df = 3, *P* = 0.053) for aphids feeding on insecticide-treated and on untreated plants (Figure 3A). However, the waveform duration per insect (WDI) of nonprobe events was significantly longer (H = 37.280; df = 3; *P* = 0.0001) on flonicamid-treated insects and those exposed to sulfoxaflor than on spirotetramat-treated and untreated plants. This particular behavior was even more remarkable in the case of flonicamid (Figure 3C). The number of passive phloem ingestion (E2) was significantly lower in the case of insects exposed to flonicamid-treated than those on spirotetramat-treated and untreated plants (H = 21.204; df = 3; *P* = 0.0001). Aphids feeding on sulfoxaflor and flonicamid-treated plants spent significantly less time on phloem sap ingestion (E2) compared with those exposed to spirotetramat-treated and untreated plants (H = 33.534, df = 3, *P* = 0.0001; Figure 3D). The percentage of probing time spent in C was significantly higher on those aphids exposed to sulfoxaflor and flonicamid than to spirotetramat-treated and untreated control plants. As a result of this, the percentage of probing time spent in E2 was significantly lower for aphids exposed to sulfoxaflor and flonicamid than to spirotetramat-treated and untreated plants (Table 2). The percentage of probing time spent in F was significantly higher on aphids exposed to sulfoxaflor than to the untreated control plants (H = 10.504, df = 3, *P* = 0.015). This suggests that aphids had plant-penetration difficulties when exposed to sulfoxaflor.

### 2.3. Evaluation of Systemic Insecticides against Transmission of Circulative Viruses by Bemisia tabaci and Myzus persicae

**Figure 4:**
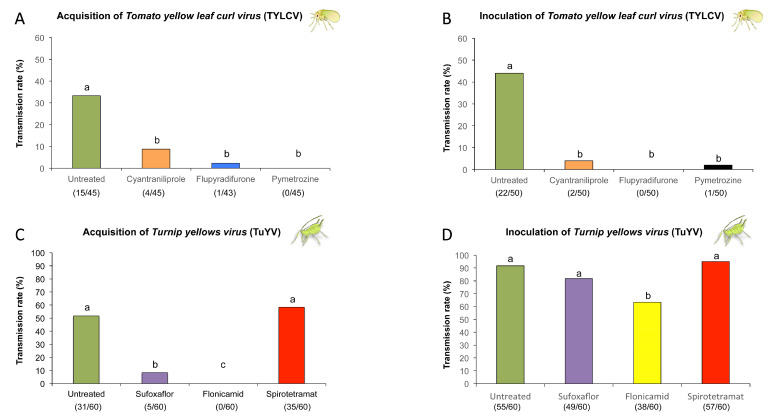
Transmission rate (%) of circulative viruses by insect vectors after exposure to insecticide-treated plants. (**A**) Transmission rate of tomato yellow leaf curl virus (TYLCV) by *Bemisia tabaci* to tomato plants after 6 h of acquisition access period (AAP) and (**B**) after 4 h of inoculation access period (IAP). (**C**) Transmission rate of turnip yellows virus (TuYV) by *Myzus persicae* to *Physalis floridana* plants after 6 h of AAP and (**D**) after 4 h of IAP. Numbers of infected plants per total number of tested plants are in parentheses.

Results of TYLCV transmission rate by *B. tabaci* to tomato plants treated with systemic insecticides (cyantraniliprole, flupyradifurone, and pymetrozine) indicated that the three tested systemic insecticides were effective in reducing both the acquisition (Figure 4A) and inoculation (Figure 4B) efficiency of TYLCV by whiteflies. The transmission rate of TYLCV by *B. tabaci* exposed to virus-infected source plants previously treated with systemic insecticides for 6 h of acquisition access period (AAP) was lower than that on untreated plants (untreated vs. cyantraniliprole: χ^2^ = 8.07, *P* = 0.005; untreated vs. flupyradifurone: χ^2^ = 14.21, *P* = 0.0001; untreated vs. pymetrozine: χ^2^ = 18, *P* = 0.0001) (Figure 4A). Furthermore, the transmission rate of TYLCV was significantly lower when viruliferous whiteflies were placed on insecticide-treated test plants for 4 h of inoculation access period (IAP) than when exposed to untreated plants (untreated vs. cyantraniliprole: χ^2^ = 21.93, *P* = 0.0001; untreated vs. flupyradifurone: χ^2^ = 28.21, *P* = 0.0001; untreated vs. pymetrozine: χ^2^ = 24.90, *P* = 0.0001; Figure 4B). No significant differences were found between the three tested insecticides. 

Spirotetramat was unable to disrupt the transmission of TuYV by *M. persicae* to *P. floridana*-plants. It failed to disrupt transmission from treated plants (χ^2^ = 0.54, *P* = 0.463; Figure 4C) and the transmission to receptor treated plants (χ^2^ = 0.54, *P* = 0.464; Figure 4D). In contrast, sulfoxaflor and flonicamid were able to significantly reduce the acquisition rate and subsequent inoculation of TuYV from insecticide-treated virus-infected source plants compared with untreated control plants (untreated vs. sulfoxaflor: χ^2^ = 26.83, *P* = 0.0001; untreated vs. flonicamid: (χ^2^ = 41.80, *P* = 0.0001; Figure 4C). Therefore, both insecticides could reduce the secondary spread of TuYV. In addition, flonicamid was able to significantly reduce (χ^2^ = 13.81, *P* = 0.0002) the inoculation rate of TuYV when viruliferous aphids were placed on treated receptor plants (Figure 4D). This result suggested that flonicamid could also interfere with the primary spread of TuYV.

## 3. Discussion

The effective management of insect vectors of plant viruses is essential for minimizing vector-borne diseases in crops [66]. Recently, some cotton lines expressing Bt toxins (MON 88702) were found to have an antifeedant effect on *Frankliniella fusca* (Hinds) that probed and ingested fewer times than those on non-Bt cotton [67]. Such alterations in the feeding behavior of thrips are known to reduce their ability to transmit tospoviruses [68]. In this way, some chemical insecticides or toxins can play an important role in effectively reducing vector populations and the spread of plant viruses. Specific chemical compounds may alter the feeding behavior of vectors in a way that transmission of phloem-restricted viruses and plant pathogenic bacteria can be disrupted [32,49]. However, insecticides fail to deter virus transmission if an insect vector can insert its mouthparts and continuously feed from vascular tissue before its death. Thus, insecticides need to act fast enough to prevent long access periods into the phloem/xylem and induce feeding cessation as fast as possible.

In the present study, we used the electrical-penetration-graph (EPG) technique as a tool to understand the feeding behavior of two sap-feeding insect pests exposed to systemic insecticides. Furthermore, we assessed their ability to reduce both the primary and secondary spread of two phloem-restricted viruses, TYLCV and TuYV, which are transmitted in a circulative manner by *B. tabaci* and *M. persicae*, respectively. Our EPG results showed no evidence of deterrence or delays in probing by any of the insect vectors when exposed to the tested insecticides, as the time elapsed from the beginning of the EPG recording until the first probe was the same for whiteflies and aphids exposed to insecticide-treated and untreated control plants. We observed that whiteflies exposed to cyantraniliprole and flupyradifurone showed fewer and a shorter duration of probes than those on untreated plants, this being even more remarkable on plants treated with flupyradifurone. Furthermore, phloem activities (salivation and phloem passive ingestion) of whiteflies were suppressed (feeding cessation) with both insecticides (cyantraniliprole and flupiradifurone). Previous studies showed similar results on the effects of pymetrozine [24] and sulfoxaflor [31] on the feeding behavior of *Myzus persicae*. Caballero et al. [53] observed that cyantraniliprole provided the excellent control of adult whiteflies. They found that this insecticide is systemic and induces rapid feeding cessation, in consequence reducing the transmission of TYLCV by *B. tabaci* [53]. This mode of action is consistent with our results, where only four out of 45 plants were infected with TYLCV after 6 h of acquisition access period (AAP) from virus-infected plants, and two out of 50 plants after 4 h of inoculation access period (IAP) to cyantraniliprole-treated plants. The reduction of the transmission rate of TYLCV was even more remarkable with flupyradifurone (one out of 43 infected plants after 6 h of AAP, and 0 out of 50 after 4 h of IAP) and pymetrozine (0 out of 45 infected plants after 6 h of AAP on treated plants, and one out of 50 after 4 h of IAP on treated plants; Figure 4). Consequently, the three tested chemical compounds showed to be valuable in reducing both the primary and secondary spread of TYLCV to tomato plants. In the same way, Roditakis et al. [57] found that only 15% of plants treated with flupyradifurone were infected by TYLCV with the high pressure of viruliferous whiteflies. Smith and Giurcanu [69] also showed that flupyradifurone was the insecticide that more efficiently reduced TYLCV infection. They also observed significant differences in the percentage of TYLCV-infected plants treated with pymetrozine when compared with untreated plants. EPG results presented in our study showed that the total duration of the salivation phase (E1) and phloem sap ingestion (E2) in pymetrozine-treated plants was not significantly different than that on untreated plants and the rest of insecticide treatments (Figure 2D). However, Civolani et al. [30] observed that pymetrozine also reduced the number and duration of phloem-related events. This discrepancy is probably related to different doses applied in both studies. Civolani et al. [30] used a dose of 250 ppm active ingredient (ai), while we used a much lower dose in our study (100 ppm ai). The mode of action of pymetrozine, which is the immediate and irreversible cessation of stylet penetration [24,70], appears to be sufficiently rapid to significantly reduce TYLCV transmission by adult whiteflies. However, Polston and Sherwood [49] suggested that pymetrozine might have some effects on plant defence mechanisms against TYLCV infection on tomato plants. This could explain why whiteflies were able to produce phloem activities associated with the transmission of TYLCV in pymetrozine-treated plants, but virus infection was significantly reduced in our study.

In the present study, we evaluated the effect of three systemic insecticides, namely, flonicamid, sulfoxaflor, and spirotetramat treatments, on the feeding behavior and transmission efficiency of TuYV by *M. persicae*. In the same way as for TYLCV, TuYV is inoculated during the phloem salivation phase (E1), and acquisition occurred during passive phloem sap ingestion (E2) [35]. Obtained results in the present study showed that aphids spent similar time in phloem salivation (E1) on plants treated with systemic insecticides than on untreated plants. This could explain why aphids were able to inoculate TuYV on plants treated with sulfoxaflor and spirotetramat after 4 h of IAP. However, the number of plants infected with TuYV was significantly reduced in plants treated with flonicamid (38 out of 60 infected test plants) after 4 h of IAP when compared with untreated plants (55 out of 60 infected plants). Therefore, our results suggested that flonicamid was the only insecticide of those tested that could reduce both the primary and secondary spread of TuYV. Flonicamid has been described as an insecticide that inhibits the feeding behavior of aphids within 0.5 h of treatment without noticeable poisoning symptoms, such as convulsion, and this antifeeding activity remains until the insect dies [64]. This fact is consistent with the long duration of nonprobing in flonicamid-treated plants (WDI: 21,299.8 ± 1582.7 s) compared with untreated plants (WDI: 5905.4 ± 977.8 s). Furthermore, the transmission rate after aphids spent 6 h of AAP on virus-infected source plants was significantly reduced on plants treated with sulfoxaflor (five out of 60 infected test plants) and flonicamid (0 out of 60 infected test plants), but no differences in transmission rate were observed between aphids exposed to spirotetramat (35 out of 60 infected plants) and the untreated control plants (31 out of 60 infected plants). The low duration of passive phloem ingestion of aphids feeding on sulfoxaflor- and flonicamid-treated plants could explain the reduced transmission efficiency of TuYV. Luteoviruses such as TuYV are acquired during the prolonged sap phloem ingestion phase (E2) while feeding on infected plants [71]. 

In summary, our work shows that specific systemic insecticides can be useful for reducing vector numbers, but can also play an important role in reducing the spread of phloem-restricted viruses by disrupting the feeding behavior of their vectors.

## 4. Material and Methods

### 4.1. Insects and Plants

A virusfree *Myzus persicae* colony was initiated from a single virginiparous female collected from a pepper plant (*Capsicum annuum* L.) in El Encín (Madrid, Spain) in 1989, and later adapted and reared on *Physalis floridana*. The aphid colony was maintained in a growth chamber at 23:18 °C (day:night), a photoperiod of 14:10 h (light:dark), light intensity of 200 µmol m^−2^ s^−1^, and relative humidity of 70%. Seven-to-ten-day-old apterous aphids were used for experiments.

A *Bemisia tabaci* (Mediterranean species, MED) colony was kindly supplied in 2007 by Enrique Moriones at La Mayora, CSIC (Málaga, Spain), and maintained at ICA-CSIC (Madrid, Spain) on eggplants (*Solanum melongena* L.) in greenhouse conditions (temperature ranges of 24:20 ± 2 °C (day:night), photoperiod of 16:8 h (light:dark), relative humidity of 70%–80%). Whiteflies were synchronized prior to experiments to guarantee age homogeneity. Eggplants with five expanded leaves were placed on metal-frame cages covered by an insectproof net with 400 whitefly adults (≈1:1 female:male) per plant. After 24 h, adult whiteflies were removed by aspiration to synchronize egg hatching and nymphal development.

*Physalis floridana* and tomato (*Solanum lycopersicum* L. var. Marmande) plants were used as virus sources and receptor plants in virus-transmission experiments. *P. floridana* and tomato plants were transplanted in 8 × 8 cm pots at the 2-leaf stage with a mixture of soil:vermiculite (2:1). Plants were watered three times a week using 20–20–20 (N–P–K) Nutrichem fertilizer (Miller Chemical and Fertilizer Corp., Pennsylvania, USA) at a dose of 1 g/L. 

### 4.2. Virus Source Plants

The TYLCV isolate used for the experiments was a TYLCV-IL type (ES:Alm:Pep:99) with GenBank accession number AJ489258, described by Morilla et al. [72]. Tomato plants were infected with TYLCV 4–5 weeks before the virus-transmission experiments started. Groups of 30 adults of *B. tabaci* were collected from the virusfree colony and placed in clip cages previously installed on young terminal leaflets of tomato plants, showing clear symptoms of TYLCV for an acquisition access period (AAP) of 72 h. Then, viruliferous insects were transferred to healthy 3–4-leaf stage tomato plants for a seven-day inoculation access period (IAP). After the IAP, each clip cage containing the leaf infested with viruliferous insects was removed from the plants to eliminate any remaining eggs and nymphs. Inoculated plants were placed in insectproof cages in a greenhouse (temperature ranges of 24:20 ± 2 °C (day:night) photoperiod of 16:8 h (light:dark), relative humidity of 70%–80%). 

An isolate TuYV-FL1 from turnip yellows virus, kindly supplied by Etienne Herrbach (INRA, France) [73], was used. *Physalis floridana* plants were infected with TuYV four weeks before the transmission experiments started. Adults of *M. persicae* exposed to an acquisition access period (AAP) of 48 h on TuYV-infected plants were used to inoculate virusfree *P. floridana* source plants. After the AAP, the aphids were transferred to healthy *Physalis* receptor seedlings (2–3-true-leaf stage) for a 72 h inoculation access period (IAP) and then removed. Receptor plants were transferred to an aphidfree growth chamber at 24:20 °C (day:night) with a photoperiod of 16:8 h (light:dark) for 3–4 weeks. 

All tomato and *P. floridana* plants inoculated with TYLCV or TuYV, respectively, were tested for the presence of the virus by double antibody sandwich enzyme-linked immunosorbent assay (DAS-ELISA) [74] using commercial antibodies (Bioreba AG, Reinach, Switzerland and Agdia Inc., Indiana, IN, US, respectively) following the manufacturer’s protocol before experiments began.

### 4.3. Insecticide Applications

Test plants were treated 24 h before the experiment began with the selected systemic insecticides and distilled water (untreated control). The recommended dose of the active ingredient (ai) used for experiments and mode of action according to the Insecticide Resistance Action Committee (IRAC) [75] are described in Table 3. All plants were sprayed until run-off (upper and lower side of the leaf) using a compression sprayer with shut-off valve and adjustable conic nozzle (Berry 1.5, Matabi, Goizper Sprayin, Gipuzkoa, Spain). 

### 4.4. Effect of Different Systemic Insecticides on Probing and Feeding Behavior of Bemisia tabaci and Myzus persicae

The probing and feeding behavior of *B. tabaci* and *M. persicae* on insecticide-treated plants was monitored using the electrical-penetration-graph (EPG) technique. Test plants were sprayed 24 h before the EPG experiments began.

Young female adults of whiteflies and apterous adults of aphids were immobilized and attached to a thin gold wire (12.5 and 18.5 µm diameter, respectively) glued to a copper wire (2 cm length) with the help of a water-based silver conductive paint (EPG-Systems, The Netherlands) following the methodologies described by Rodriguez-López et al. [76] for whiteflies, and those of Garzo et al. [31] for aphids. Then, insects were connected to the EPG device and placed on the abaxial side of a young leaf where they were allowed to probe and feed for eight hours.

A Giga-8 DC-EPG device with 1 GΩ (EPG Systems, The Netherlands) resistance was used to monitor the probing and feeding activities of insects on insecticide-treated and untreated control plants. EPG signals were acquired and analyzed using Stylet+ software for Windows (EPG Systems, The Netherlands). A minimum of 15 recordings were made for each treatment using a different single aphid and plant combination for each replicate. The output given by the Sarria et al. [77] workbook for each given insect (replicate) was used for calculating the treatment mean for each EPG sequential and nonsequential variable (raw data are provided in Supplementary File S1). Selected EPG variables (mean ± SE) were calculated and compared between treatments as described by Backus et al. [78]. Proportion of individuals that produced the waveform type (PPW); and number of waveform events per insect (NWEI), which was calculated as the sum of the number of events of a particular waveform divided by the total number of insects under each treatment. Waveform duration per insect (WDI) was calculated as the sum of durations of each event of a particular waveform made by each insect that produced that particular waveform divided by the total number of insects under each treatment. If there were no events of a particular waveform, it was scored as 0. 

### 4.5. Evaluation of Activity of Systemic Insecticides against Transmission of Circulative Viruses by Bemisia tabaci and Myzus persicae

Preliminary experiments were carried out to determine the ideal number of insects, and the optimal AAP and IAP needed to obtain effective transmission in the control treatments and run statistically meaningful comparisons between insecticide-treated and untreated plants.

All tomato and *P. floridana* receptor plants were tested for the presence of TYLCV or TuYV by DAS-ELISA four weeks after the transmission experiments were completed.

#### 4.5.1. Can Insecticides Deter Acquisition and Subsequent Transmission from Insecticide-Treated Plants?

The aim of these experiments was to assess if the selected systemic insecticides can reduce the secondary spread of circulative viruses. For such purpose systemic insecticides were applied to a virus-infected plant to assess if nonviruliferous insect vectors that land on an infected plant can acquire and transmit the virus to a noninfected receptor plant.

The virus source plants (either TYLCV or TuYV infected plants) were treated with the selected systemic insecticides at the recommended dose (Table 3) 24 h before the transmission experiments started. Water treated plants were used as untreated control. Once the products became systemic after 24 h, nonviruliferous insects were exposed to the virus-infected source plants for an acquisition access period (AAP) of 6 h. Then, insects were transferred (4 whiteflies/test plant or 10 aphids/test plant) to untreated virus free receptor plants and left for a 72 h inoculation access period (IAP). Then, receptor plants were sprayed with imidacloprid at 200 ppm ai to remove all the whiteflies and aphids. A total of 45 and 60 receptor plants were used for each treatment for whiteflies and aphids, respectively. All the insects that were exposed to insecticides on virus-infected source plants remained alive after the AAP.

#### 4.5.2. Can Insecticides Deter Transmission to Insecticide-Treated Plants? 

The aim of these experiments was to assess whether systemic insecticides could reduce the primary spread of circulative viruses. The experiments were designed to assess if the selected compounds could avoid or reduce the transmission rate of viruliferous insects that land on healthy plants that were previously treated with systemic insecticides.

Whiteflies were exposed to TYLCV-infected untreated plants for an AAP of 48 h. Newly born aphids were reared on TuYV-infected *P. floridana* plants. Then, viruliferous insects were transferred to noninfected receptor plants previously treated with the systemic insecticides at the recommended dose (Table 3). Water-treated plants were used as untreated control. Insects remained on the test plants (4 whiteflies/test plant and 3 aphids/test plant) for an IAP of 4 h; afterwards, plants were sprayed with imidacloprid at 200 ppm ai to remove all whiteflies and aphids, and avoid further virus spread. A total of 50 and 60 test plants were used for each treatment for whiteflies and aphids, respectively.

### 4.6. Statistical Analysis 

To analyze the impact of insecticides on insect probing and feeding behavior, we used the nonparametric Kruskal–Wallis test followed by Dunn’s test with Bonferroni correction for specific pairwise comparisons. Statistical analysis was performed using IBM SPSS Statistics, version 26.0 (IBM Corp. Armonk, NY).

A chi-squared test, and Fisher’s exact test if expected values were lower than 5 (Statview II, [79]), were used to analyze the effects of systemic insecticides on virus-transmission rate. Similarly, the proportion of individuals that produced a given waveform type (PPW) was compared among the different treatment groups using a chi-squared test.

## 5. Conclusions

The obtained results indicated that the antifeeding and feeding cessation effects produced by some of the studied systemic insecticides play an important role in reducing the acquisition and inoculation rate of turnip yellows virus (TuYV) and tomato yellow leaf curl virus (TYLCV), both transmitted in a circulative persistent manner by *Myzus persicae* and *Bemisia tabaci*, respectively. Accordingly, cyantraniliprole, flupyradifurone, and pymetrozine proved to be equally effective in reducing the acquisition and inoculation of TYLCV. Therefore, these insecticides would be able to reduce, but not totally avoid, the primary and secondary spread of TYLCV. Both sulfoxaflor and flonicamid have antifeeding effects on *M. persicae*, reducing phloem sap ingestion, which, overall, limits the probability for virus acquisition from the phloem when feeding on TuYV-infected plants. Only flonicamid was able to reduce the inoculation of TuYV to treated plants, thereby potentially reducing both the primary and secondary spread of the virus. Aphids exposed to spirotetramat behaved in a similar way as those exposed to the untreated control, showing no antifeeding nor antiappetitive response. Consequently, spirotetramat failed to interfere with the transmission of TuYV by *M. persicae*.

## Figures and Tables

**Table 1:** EPG variables of probing and feeding behavior of *Bemisia tabaci* exposed to insecticide-treated and untreated plants. Values represent mean ± standard error of sequential variables and percentage of probing time spent in a specific waveform (time expressed in seconds).

Sequential Variables	Untreated Control *n* = 15	Cyantraniliprole *n* = 15	Flupyradifurone *n* = 15	Pymetrozine *n* = 15	*P*
Time to first probe from EPG start	415.6 ± 78.7 a	341.4 ± 86.3 a	311.4 ± 83.6 a	385.7 ± 105.1 a	0.766
Time from first probe to 1st E	18,861.4 ± 2714.7 b	28,458.5 ± 86.3 a	28,488.6 ± 83.6 a	22,438.1 ± 2016.8 ab	0.003
Time from the beginning of that probe to first E	921.00 ± 202.8 a	-	-	1390.5 ± 290.4 a	0.101
Time from the beginning of that probe to first E2	1091.1 ± 232.2 a	-	-	1062.5 ± 187.3 a	0.758
**Indices**					
Probing % spent in C	88.34 ± 3.1 b	99.26 ± 0.7 a	98.18 ± 1.8 a	84.13 ± 4.37 b	0.000
Probing % spent in G	5.82 ± 2.3 a	0.74 ± 0.7 b	1.82 ± 1.8 b	11.02 ± 3.6 a	0.005
Probing % spent in E1	0.28 ± 0.2 a	0 b	0 b	0.11 ± 0.0 a	0.0001
Probing % spent in E2	5.56 ± 1.9 a	0 b	0 b	4.74 ± 2.7 ab	0.0001

*P*-values were recorded according to the Kruskal-Wallis test. Mean values within a row followed by different letters were significantly different (*P* ≤ 0.05) according to Dunn’s test with Bonferroni correction.

**Table 2:** EPG variables of probing and feeding behavior of *Myzus persicae* adults exposed to insecticide-treated and untreated plants. Values represent mean ± standard error values of sequential variables, and percentage of probing time spent in a specific waveform (time expressed in seconds).

Sequential Variables	Untreated Control *n* = 18	Sulfoxaflor *n* = 19	Flonicamid *n* = 17	Spirotetramat *n* = 19	*P*
Time to first probe from start of EPG	267.1 ± 122.6 a	196.4 ± 76.2 a	1064.2 ± 558.6 a	213.7 ± 70.5 a	0.804
Time from first probe to first E	3897.7 ± 743.5 a	7625.9 ± 2029.2 a	8162.1 ± 2697.2 a	6528.1 ± 1142.6 a	0.303
Time from beginning of that probe to first E1	679.2 ± 71.8 a	1747.8 ± 610.7 a	784.8 ± 65.4a	1124.3 ± 353.1 a	0.307
Time from beginning of that probe to first E2	765.4 ± 04.9 b	1800.5 ± 665.9 b	824.3 ± 79.7 b	1233.5 ± 351.5 a	0.0001
**Indices**					
Probing % spent in C	26.36 ± 5.0 b	47.85 ± 4.3 a	48.45 ± 6.5 a	23.59 ± 1.6 b	0.0001
Probing % spent in F	10.07 ± 2.9 b	35.38 ± 5.2 a	20.26 ± 7.3 ab	18.73 ± 2.2 ab	0.015
Probing % spent in E1	5.35 ± 1.2a	5.53 ± 1.5 a	6.57 ± 1.7 a	5.26 ± 0.6 a	0.999
Probing % spent in E2	57.65 ± 6.7 a	10.00 ± 2.9 b	24.42 ± 8.1 b	50.48 ± 3.7 a	0.0001

*P*-values were recorded according to Kruskal–Wallis test. Mean values within a row followed by different letters were significantly different (*P* ≤ 0.05) according to Dunn’s test with Bonferroni correction.

**Table 3:** Systemic insecticides tested in laboratory experiments against *Bemisia tabaci* and *Myzus persicae.* *, Mode of action according to Insecticide Resistance Action Committee (IRAC) classification.

***Bemisia tabaci***					
**Active Ingredient**	**Dose (ai)**	**Chemical Class**	**Commercial Product**	**Company**	IRAC *
Cyantraniliprole	150 ppm	Ryanoid	Cyazypyr^TM^ 10%	Dupont Corporation	28
Flupyradifurone	150 ppm	Butenolides	Sivanto^TM^ 200SL	Bayer	4
Pymetrozine	100 ppm	Pyridine azomethine	Plenum^®^50% (WG) P/P	Syngenta	9
***Myzus persicae***					
**Active Ingredient**	**Dose (ai)**	**Chemical Class**	**Commercial Product**	**Company**	IRAC *
Spirotretamat	75 ppm	Ketoenols	Movento® 150 O-TEQ 15% [OD] P/V	Bayer	23
Flonicamid	60 ppm	Pyridinocarboxamide	Carbine^TM^ 50WG	FMC Corporation	29
Sulfoxaflor	24 ppm	Sulfoximines	Isoclast^TM^ active 30%	Dow Agrosciences	4

## Supplementary Materials

The following are available online at https://www.mdpi.com/2223-7747/9/7/895/s1, raw data of EPG variables and proportion of insects that produced the waveform type (PPW) are accessible as Supplementary File S1.
